# *Plasmodium falciparum* community prevalence and health-seeking behaviours in rural Sussundenga District, Mozambique

**DOI:** 10.1186/s12936-022-04326-z

**Published:** 2022-10-28

**Authors:** Dominique E. Earland, Albino Francisco Bibe, Anísio Novela, João Ferrão, Kelly M. Searle

**Affiliations:** 1 School of Public Health, University of Minnesota, Minneapolis, MN USA; 2 Escolá Secondaria de Sussundenga, Sussundenga, Manica, Mozambique; 3 Direcção Distrital de Saúde de Sussundenga, Manica, Mozambique; 4 Universidade Aberta ISCED, Beira, Mozambique

**Keywords:** Health-seeking behaviors, Malaria prevalence

## Abstract

**Background:**

Impacts of nationally directed malaria control interventions hinge on understanding malaria transmission and prevention at the community level. The decision to seek care or health-seeking behaviours provide valuable insight on knowledge of malaria, access to care, and efficacy of malaria case management. Thus far, few studies have focused on central Mozambique. The aim was to describe community level *Plasmodium falciparum* prevalence and health-seeking behaviours among residents of Sussundenga, Mozambique, a rural village in Manica Province with high malaria incidence reported at the Sussundenga-Sede health centre (RHC).

**Methods:**

A cross-sectional community-based survey was conducted from December 2019 to February 2020. A random household sampling method was used, based on enumerated households from satellite imagery. All consenting participants completed a survey about malaria risk, prevention, and health-seeking behaviours, and received a *P. falciparum* malaria rapid diagnostic test (RDT).

**Results:**

The study enrolled 358 individuals from 96 households. The *P. falciparum* prevalence was 31.6% (95% CI [26.6–36.5%]). Ninety-three percent of participants reported using the Sussundenga-Sede RHC for healthcare. Sixty-six percent of participants (N = 233) experienced at least one malaria symptom in the past month, with self-reported fever most frequently reported (19.3%). Of these, 176 (76.5%) sought care in a health facility and 174 (79%) received an RDT with 130 (63%) having a positive test. Of those with a positive RDT, 127 (97%) received artemether-lumefantrine. Following treatment, 123 (97%) participants’ symptoms resolved within a median of 3 days (IQR: 3–5) ranging from 2 to 14 days. In this high transmission setting, a high proportion of participants recognized malaria related symptoms then received a proper diagnostic test and treatment in a health facility.

**Conclusions:**

Future interventions that leverage this health-seeking behaviour and strengthen health systems for community interventions will improve malaria control and inform the efficacy of potential interventions at this particular international border.

## Background

The globally estimated 241 million cases of malaria disproportionately impact sub-Saharan Africa [1]. Mozambique was one of 29 countries that claimed 96% of global malaria cases. This high burden country reported 8,921,081 malaria cases and 1114 deaths in 2018, which represented approximately 4% of cases and deaths globally [2]. Mozambique has eleven provinces and based on data from the most recent Malaria Indicator Survey (MIS) determined that Cabo Delgado, Nampula, and Manica Provinces have the highest malaria prevalence for children under five [3]. Manica Province, unlike the other high transmission provinces, is located within the central region.

Manica Province shares a border with Tete, Sofala, Inhambane, and Gaza Provinces as well as Zimbabwe. Manica Province in comparison to the surrounding border regions had a higher malaria burden. Based on 2018 MIS data, the malaria prevalence for children under five for Manica Province was 19% higher than Tete and Sofala Provinces [3]. During 2018, the World Health Organization (WHO) reported malaria deaths were five times higher in Mozambique compared to Zimbabwe [1]. However, previous epidemiological studies that evaluated malaria control measures have not focused on the central region of Mozambique, particularly Manica Province located outside of Chimoio City [4, 5]. Manica Province had 821,775 malaria cases in 2019 [6]. Intermittent preventive treatment in pregnancy (IPTp) was available in Manica Province with 49% of pregnant women receiving at least three sulfadoxine-pyrimethamine doses [3]. In Manica Province, 87% of households had at least one insecticide-treated nets (ITNs) [3].

Sussundenga District was one of nine districts in Manica Province. This district contains 14 different RHCs and had a documented high annual incidence of malaria cases [3]. ITNs were the main preventive measure accessible to most households [3]. In Sussundenga, prompt diagnosis and treatment with artemether-lumefantrine at an RHC was the standard of care for uncomplicated malaria illness. There were recent plans to expand the use of community health workers (CHWs) for integrated community case management (iCCM) throughout Mozambique in the coming years [7].

The primary objective of this analysis was to describe community-level *P. falciparum* prevalence and health-seeking behaviours in rural Sussundenga village in Manica Province, Mozambique. This analysis was part of a pilot study, which sought to measure the community malaria prevalence, perceptions of malaria risk, utilization of malaria prevention, and health-seeking behaviour patterns to inform future malaria control efforts. The pilot study was performed in partnership with Sussundenga RHC, Sussundenga District Ministry of Health, and community leaders.

**Table 1:** Sociodemographic characteristics among malaria positive and negative individuals

	Malaria negative	Malaria positive	95% CI
	N(%)	N(%)	
Age			P < .001
< 5 years	23(9.8)	19(17.9)	(10.6–25.23)
5–10 years	33(14.1)	28(26.4)	(18.0–34.8)
10–15 years	28(12.0)	20(18.9)	(11.4–26.3)
16–20 years	43(18.4)	16(15.1)	(8.28–21.9)
20–30 years	48(20.5)	10(9.4)	(3.87–15)
> 30 years	59(25.2)	13(12.3)	(6.02–18.5)
Sex			P = .84
Male	99(43.2)	47(44.3)	
Female	130(56.8)	59(55.7)	
Employment			P = .39
Farmer	38(56.7)	17 (81)	
Miner	1(1.5)	0(0)	
Health worker	1(1.5)	0(0)	
Teacher	15(22.4)	1(4.8)	
Coroner	1(1.5)	0(0)	
Business owner	11(16.4)	3(14.2)	
Education			P < .01
Grades 1–5	8(7.5)	14(6)	(3.00–9.18)
Grades 6–7	8(7.5)	30(12.8)	(8.69–17.4)
Grades 8–10	17(16)	61(26.1)	(20.8–32.2)
Grades 11–12	9(8.5)	40(17.1)	(12.5–22.3)
University	0(0)	2(2.6)	(0–2.07)
N/A	64(60.5)	83(35.4)	(29.9–42.3)
Bed net usage			P < .01
No	72(30.9)	50(46.3)	(36.9–55.7)
Yes	161(69.1)	58(53.7)	(44.3–63.1)
Regular Health Center			P = .33
Sussundenga-Sede	215(91.9)	105(97.3)	
Munhinga	3(1.3)	1(0.9)	
Dombe	0(0)	1(0.9)	
Muôha	1(0.4)	0(0)	
Rotanda	1(0.4)	0(0)	
Other	14(6)	1(0.9)	

## Methods

### Study area

**Fig. 1:**
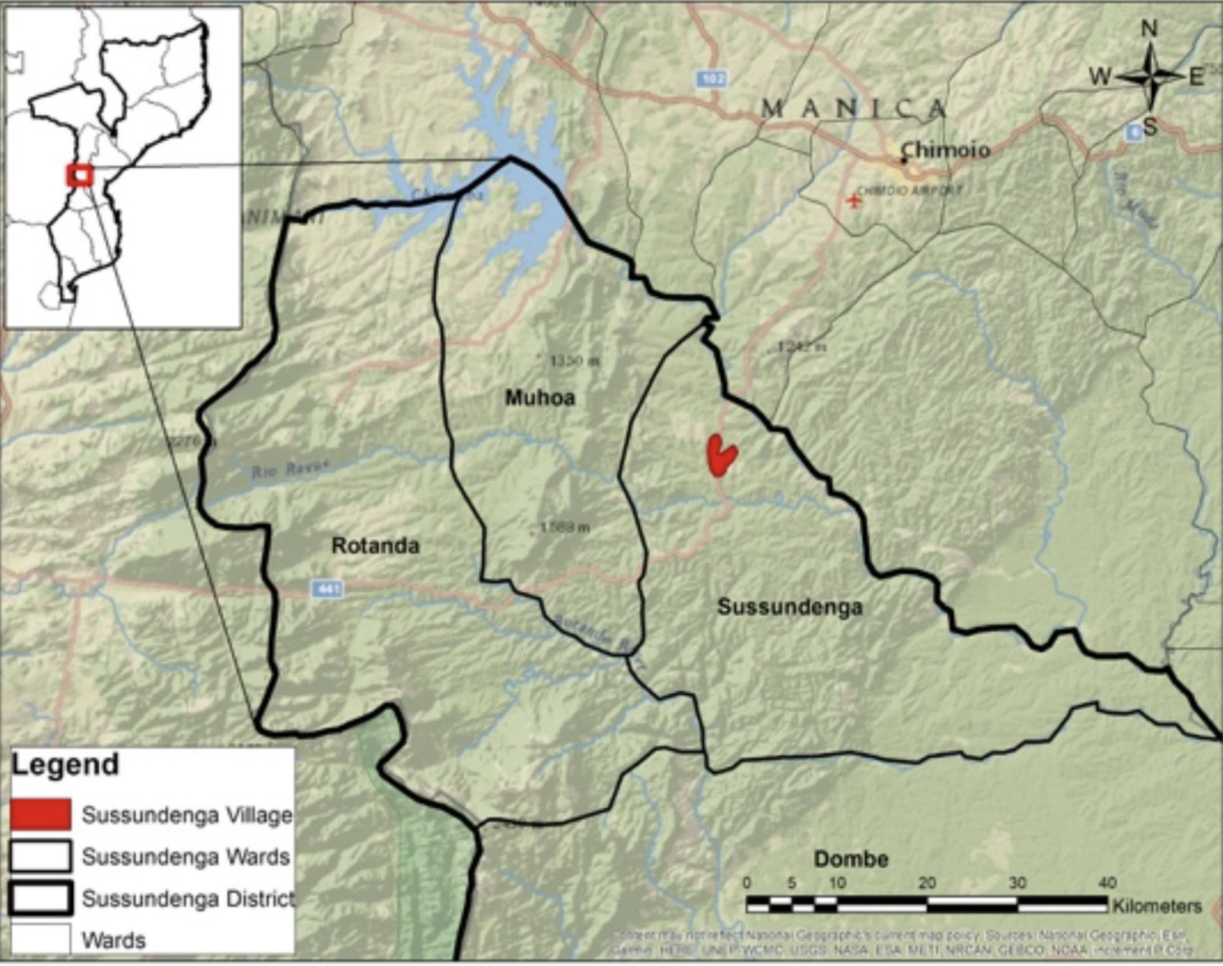
Map of Sussundenga catchment area

The survey was conducted in Sussundenga-Sede catchment area, a region within Sussundenga district. This is approximately 42 km from the Provincial capital of Chimoio city in central Mozambique (Fig. 1). Sussundenga District is a rural agrarian community that shares a border with Zimbabwe. The district had area of 7017 km^2^ and the district population was 171,056 residents [8]. From September to March, Manica Province has high temperatures and increased rainfall which contributes to the reported seasonally high malaria incidence. The main rural health centre (RHC) in the village was the Sussundenga-Sede health centre, with 14 smaller RHCs located in the district. The study sampled households from Sussundenga District because of the high incidence, proximity to the Zimbabwe border, and accessibility to several RHCs. The Mozambican National Malaria Control Programme initiated a national ITN campaign from 2019 to 2020 to improve coverage [6]. ITNs and IPTp were accessible to Sussundenga District residents through the antenatal care centres, while indoor residual spraying (IRS) became available to central districts in 2018.

### Study design and data collection

**Fig. 2:**
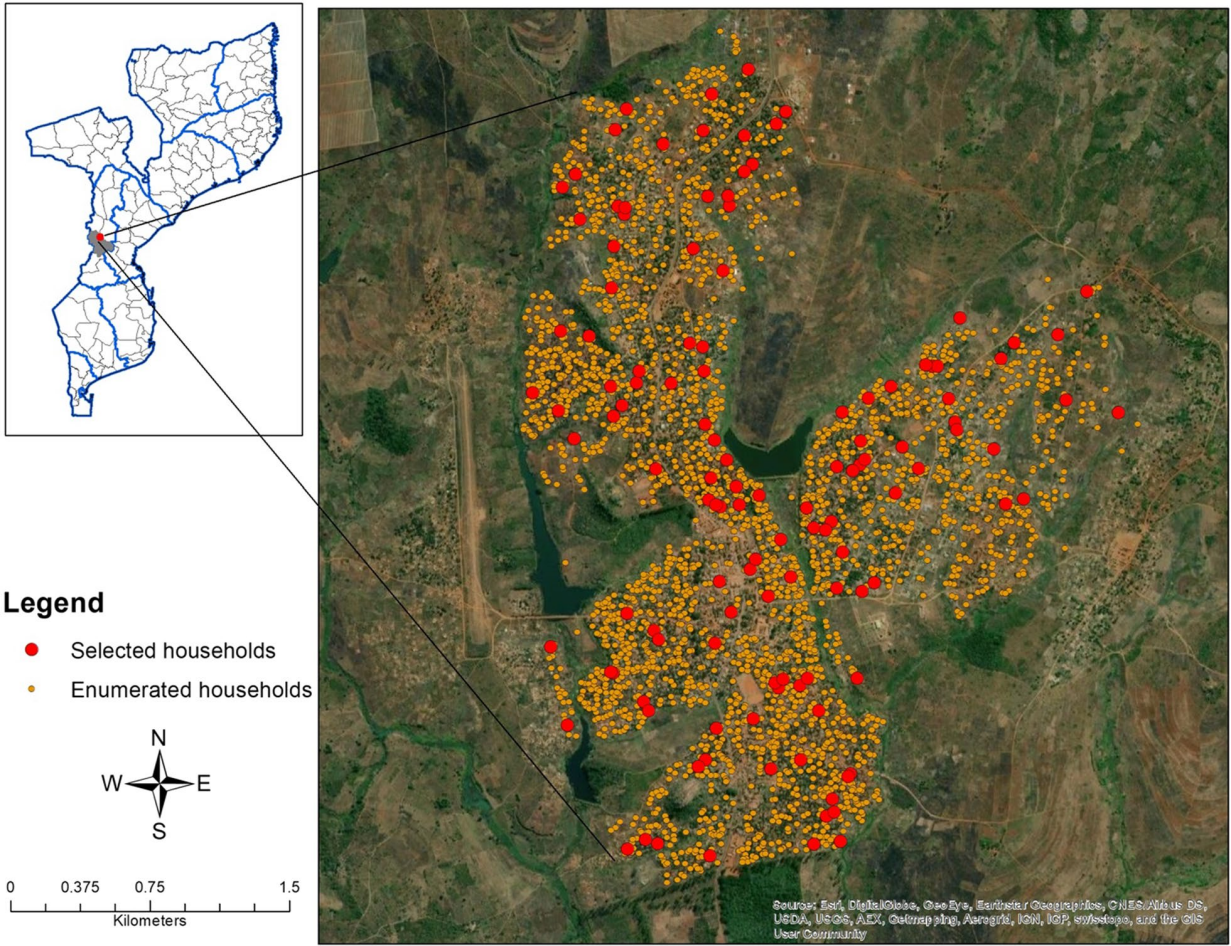
Map of enumerated and selected households

A cross-sectional community-based survey was administered from December 2019 to February 2020 in Sussundenga village. This analysis focused on health-seeking behaviours data from a larger survey for the pilot study which evaluated malaria risk based on sociodemographic, environmental, migration, housing construction, and other risk factors. Satellite imagery was used to enumerate 2889 households in Sussundenga catchment area (Fig. 2). To account for potential misclassification of household structures and refusals, a random sample of 125 households were selected for screening from satellite imagery. Data collectors used GPS coordinates to approach selected households for participation and enrolled 100 households. The household size assumption was an estimated 5–6 total residents per household at the time of survey administration. Data collectors determined eligibility through a notification visit with the head of the household and assigned each household member a unique identifier. As a pilot study the sample size was determined to estimate the community prevalence and detect differences in specific risk factors between those with and without *P. falciparum* infection measured by RDT.

Data collectors also obtained informed consent for all adult residents and parent/guardian permission for children between 3 months and 12 years old and assent for minors between 13 and 17 years old. Enrollment eligibility criteria was any full-time resident older than 3 months. After the notification visit, data collectors administered the electronic survey and recorded household GPS coordinates on a tablet computer using a REDCap^®^ (Research Electronic Data Capture) mobile application. All participants present at the time of survey administration who were older than 12 years old completed the survey and parents provided responses for children 3 months old to 12 years old.

A study nurse collected a finger prick blood sample and administered a malaria rapid diagnostic test (RDT) [Right Sign Malaria Pf (Biotest, Hangzhou Biotest Biotech Co, China]. All participants with positive results were referred to Sussundenga-Sede RHC for confirmation of diagnosis and treatment. All symptoms reported to data collectors were self-reported based on malaria cases that occurred in the previous month. All data were collected and stored using the REDCap^®^ server hosted at University of Minnesota School of Public Health and was treated confidentially [9, 10]. Ethical review and approval for the study was completed by the Institutional Review Board (IRB) at the University of Minnesota [STUDY00007184] and from A Comissão Nacional de Bioética em Saúde (CNBS) at the Ministry of Health of Mozambique [IRB00002657].

#### Data analyses

All data analyses were performed using R (version 4.1.1). In this analysis, individuals older than 12 years old were adults and individuals 12 years old and younger were children. This cut-off was determined by the age at which individuals transition from pediatric to adult care. Children less than 13 years old were not included in the education level and malaria prevalence analysis to understand the association among adults. Community *P. falciparum* prevalence was determined by RDT results at the time of the survey. Pearson Chi square tests were used to compare the socio-demographic characteristics between malaria positive and negative individuals. Age, sex, employment, education, bed net usage, and regular health centre usage were compared between those with positive and negative RDT results without adjusting for covariates.

Health-seeking behaviours were described as proportions with 95% confidence intervals. The variables to describe the care continuum included care seeking with malaria symptoms, diagnosis, and treatment. All health-seeking behaviour variables were nominal categorical variables in the survey. The primary health-seeking and RDT malaria diagnosis variables were among all participants who in the past month reported at least one of the following malaria symptoms: fever, headache, chills, vomiting, nausea, diarrhoea, cough, joint pain, or body aches. In the survey, participants reported where they first went to retrieve medication to treat their malaria symptoms which could include a RHC, informal shop, relative or friend, and others.

The primary health-seeking variable was defined as the proportion of individuals that sought care at an RHC among participants who in the past month reported malaria symptoms. In the survey, participants reported malaria diagnosis by RDT, smear, or none performed. The RDT malaria diagnosis variable was defined as the proportion of individuals who received a RDT among participants who in the past month reported malaria symptoms. In the survey, participants reported use of various malaria medications including traditional medicine, paracetamol, and other anti-malarial drugs. The received treatment variable was defined as the proportion of participants who reported artemether-lumefantrine treatment among participants who reported a positive malaria diagnosis by RDT within the past month.

Symptom resolution was defined as malaria positive individuals who received treatment that reported symptom resolution. The proportion of participants that reported a resolution of their symptoms after completing prescribed treatment was calculated. The time to symptom resolution after treatment described treatment compliance and prompt symptom resolution, which suggested participants completed the treatment dosage and experienced parasite clearance. The days until symptoms resolved variable was a continuous variable that was reported based on an individual’s most recent malaria infection. A violin plot was created to compare the number of days until symptoms resolved by all participants, adults, and children.

## Results

### Socio-demographic characteristics

The study enrolled 96 households with 358 individual household members who were present at the time of the study visit (Table 1). The age range was 1–80 years and children aged 1–12 years old represented 36% of participants. The malaria prevalence in the study population was 31.6% (95% CI [26.6–36.5%]). There were no gender differences in malaria prevalence (p = 0.84). There were statistically significant differences in malaria prevalence by age (p < 0.001). The highest malaria prevalence was among children aged 5–10 years old (26.4% [95% CI 18.0–34.8]). There were statistically significant differences in malaria prevalence by level of education (p < 0.01). The highest malaria prevalence was among adults who received education up to grades 8–10 (26.1% [95% CI 20.8–32.2%]). Reported ITN use was moderate with 65% of participants reporting sleeping under an ITN the previous night. There were not statistically significant differences in ITN use by age. ITNs were protective among those who reported use the previous night, with 26.5% (95% CI [20.1–32.9%]) prevalence among those who did report usage compared to 41.0% (95% CI [32.3–50.3%]) prevalence among those who did not report ITN use the previous night (p < 0.01). There were not statistically significant differences in malaria prevalence by occupation, although farmers had the highest malaria prevalence compared to other occupations. Ninety-three percent (93%) of participants reported using the Sussundenga-Sede RHC for their last episode of malaria symptoms.

#### Health-seeking characteristics and timeline

**Fig. 3:**
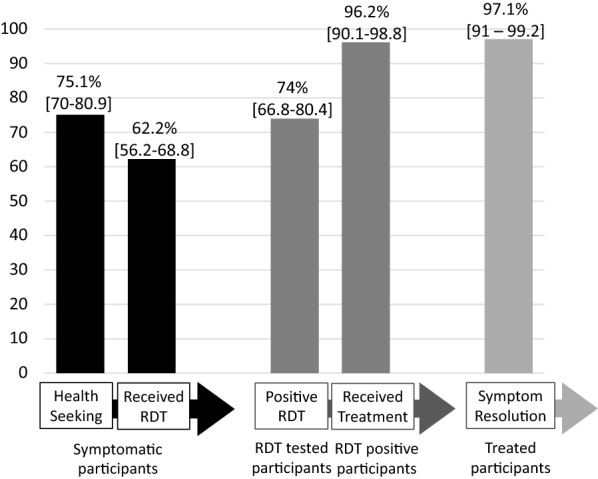
Health seeking among symptomatic, RDT positive, and artemether lumefantrine treated participants

Sixty-six percent (66%) of participants (N = 233) reported at least one malaria related symptom in the past month, with fever most frequently reported (19.3%). Of the participants that reported symptoms, 75.1% (95% CI [70.0–80.9%]) sought care at a health and 62.2% (95% CI [56.2–68.8%]) received an RDT. Among all participants tested with an RDT, 74.0% (95% CI [66.8–80.4%]) were malaria positive. Of the RDT positive participants, 96.2% (95% CI [90.1–98.8%]) reported receiving artemether lumefantrine, the standard of care for uncomplicated malaria in Mozambique, and 97.1% (95% CI [91.0–99.2%]) of participants treated with artemether-lumefantrine reported symptom resolution after completing the treatment. Of the four participants that reported not receiving artemether-lumefantrine, one participant reported having severe malaria and received quinine, and the remaining participants had not reported receiving the standard of care (see Fig. 3).

Three participants (2.9%), which were school-aged children, reported unresolved symptoms after receiving the standard of care at the Sussundenga-Sede RHC (diagnosis with an RDT and treatment with artemether-lumefantrine). Of those three participants, one continued to be malaria positive at the time of the survey. Four (3.3%) participants had a malaria related hospitalization after reported symptoms within the last month. Two of the individuals with reported severe disease requiring hospitalization were adults older than 40 years and the other two individuals were children. All of the malaria related hospitalizations reported seeking care, received treatment other than standard of care, and required additional treatment. Three of the four hospitalized individuals reported symptom resolution.

#### Efficacy and access to care

**Fig. 4:**
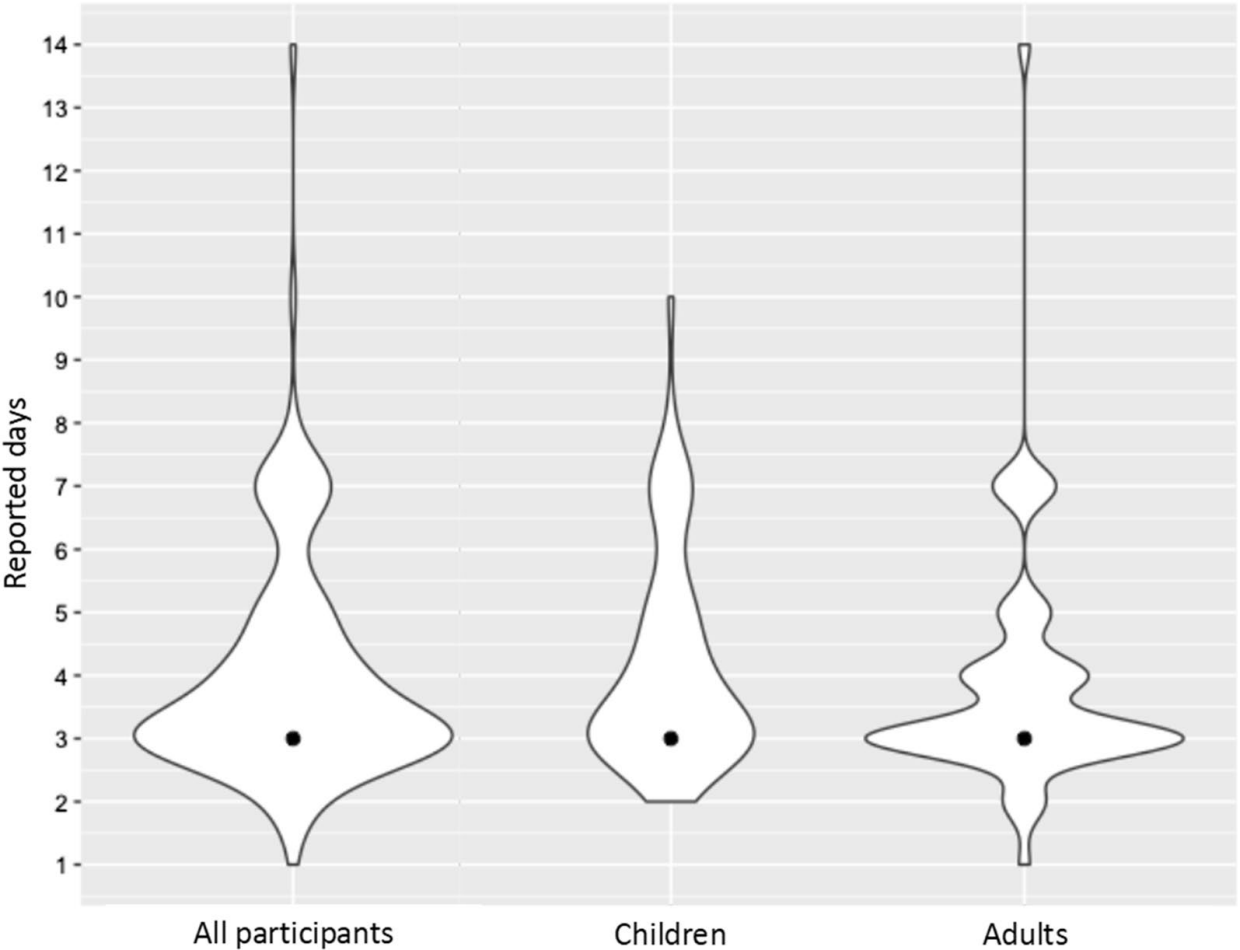
Reported days until symptoms resolved amongst participants that sought care

Median time until reported symptoms resolved was 3 days (IQR: 3–5) and ranged from 2 to 14 days. Figure 4 shows the distribution of reported days until reported symptoms resolved among all participants, children, and adults. There were no significant differences in median days until symptoms resolved between children and adults. Adults had a greater probability of having reported symptoms resolve in 3 days compared to children. The distribution of reported days until symptoms resolved was bimodal for children compared to a multimodal distribution among adults. Nearly all (97.7%) participants reported preference for care at the Sussundenga-Sede RHC, the majority reported this preference because it was close to their home (94.8%).

## Discussion

In this study, the Sussundenga community *P. falciparum* prevalence was highest among children aged 5–10 years old compared to other ages. Findings from this study indicate a high degree of treatment seeking behaviours among children and adults which led to prompt reported symptom resolution, specifically at the Sussundenga-Sede rural health centre. The high level of health-seeking behaviours could be explained by the close proximity of the largest rural health centre in the village, as several studies have demonstrated the association of shorter travelling distance and utilization of malaria healthcare [11–13].

The 2018 Mozambique Malaria Indicator Survey (MIS) similarly reported a high malaria prevalence in Manica Province among children under five (39%), the third highest in Mozambique [3]. In the study, 65% of participants reported use of an ITN the previous night, which is similar to Manica Province data from MIS (68.8%). Although a high proportion of participants who reported a malaria positive diagnosis received proper treatment and reported symptom resolution, fewer participants who reported malaria symptoms sought care at an RHC and received an RDT. Data from the 2018 MIS reported from households in Manica Province, determined 60% of individuals with fever sought treatment at a RHC and 39.2% of individuals with fever received a RDT. In the study, reported health-seeking at a RHC and reported RDT testing among participants who reported malaria symptoms was higher than 2018 MIS Manica Province data.

National Malaria Control Programme interventions that promote malaria social and behavioural change could improve initiation of treatment seeking behaviours in Sussundenga District, leading to a greater proportion of symptomatic individuals to be tested [6]. Expansion of the thousands of community health workers (CHWs) within the national integrated community case management (iCCM) strategy could increase access to treatment, diagnostics, and ITNs [3, 13]. The study demonstrated persistent health-seeking behaviours among households, suggesting a high degree of malaria symptom recognition. Possible interventions like utilizing CHWs could be effective to close existing gaps in testing and access to treatment [14]. Additional resources for CHWs to deliver ITNs and treat asymptomatic malaria cases could reduce transmission in high burden settings like Sussundenga district [15–17].

There were several limitations to this study, the first being the cross-sectional design which hindered observing the potential for changing health-seeking behaviours over time. The study relied on self-reported metrics and lacked information directly from RHCs. Additional understanding of RHC diagnostic and treatment policy compliance as well as patient treatment compliance could further explain our findings [16]. Yet, with self-reported responses there is the potential that recall bias could have impacted the accuracy of responses about information surrounding previous malaria symptoms and care.

Prior studies in Manica province determined rural regions have higher prevalence in comparison to urban areas [3], but have not yet quantified the community prevalence in the majority of these rural regions. Few studies have determined the community prevalence of regions in Sussundenga district, especially Sussundenga village. The study found households promptly sought care to treat their malaria symptoms, which suggests that the RHCs and individual households are strengths to incorporate into future studies and interventions. Efforts to understand the drivers of community prevalence in well-resourced, high burden areas could inform innovative community driven malaria prevention and vector control measures.

## Conclusion

In this study, a majority of participants who reported malaria diagnosis by RDT received treatment at a health facility and had symptom resolution. The high community prevalence in Sussundenga District could be driven by reduced treatment seeking upon malaria symptom development, malaria diagnosis by RDT, and ITN utilization. This suggests additional prevention methods like increasing ITN coverage and CHWs to improve malaria treatment seeking and diagnosis are necessary to address the malaria burden in the district.

## Data Availability

All data generated or analysed during this study are included in this published article.

## Abbreviations

CHW: Community Health Workers
iCCM: Integrated community case management

## References

[1] WHO (2021). World malaria report 2021.

[2] Mozambique: Country Profiles. Geneva: World Health Organization. 2019. https://www.who.int/gho/countries/moz/country_profiles/en/. Accessed 1 June 2022.

[3] Instituto Nacional de Saúde/INS, Instituto Nacional de Estatistica/INE, Programa Nacional de Controlo da Malaria/PNCM, ICF. Mozambique Inquérito Nacional sobre Indicadores de Malaria (IIM)2018. Maputo, Mozambique: INS/Mozambique, INE, PNCM, ICF. 2019. http://dhsprogram.com/pubs/pdf/MIS33/MIS33.pdf. Accessed 1 June 2022.

[4] Aide P, Candrinho B, Galatas B, Munguambe K, Guinovart C, Luis F (2019). Setting the scene and generating evidence for malaria elimination in Southern Mozambique. Malar J.

[5] Plucinski MM, Candrinho B, Chambe G, Muchanga J, Muguande O, Matsinhe G (2018). Multiplex serology for impact evaluation of bed net distribution on burden of lymphatic filariasis and four species of human malaria in northern Mozambique. PLoS Negl Trop Dis.

[6] Relatório do Primeiro Semestre 2020. Programa Nacional de Controlo da Malaria. Direcção Provincial de Saúde de Manica; 2020.

[7] Chilundo BG, Cliff JL, Mariano AR, Rodríguez DC, George A (2015). Relaunch of the official community health worker programme in Mozambique: is there a sustainable basis for iCCM policy?. Health Policy Plan.

[8] Instituto Nacional de Estatística. Anuário Estatístico, Província de Manica 2017. Província de Manica. 2018. http://www.ine.gov.mz/estatisticas/publicacoes/anuario/provincia-de-manica. Accessed 1 June 2022.

[9] Harris PA, Taylor R, Thielke R, Payne J, Gonzalez N, Conde JG (2009). Research electronic data capture (REDCap)—a metadata-driven methodology and workflow process for providing translational research informatics support. J Biomed Inform.

[10] Harris PA, Taylor R, Minor BL, Elliott V, Fernandez M, O’Neal L (2019). The REDCap consortium: building an international community of software platform partners. J Biomed Inform.

[11] Cassy A, Saifodine A, Candrinho B, Martins MDR, da Cunha S, Pereira FM (2019). Care-seeking behaviour and treatment practices for malaria in children under 5 years in Mozambique: a secondary analysis of 2011 DHS and 2015 IMASIDA datasets. Malar J..

[12] Treleaven E, Whidden C, Cole F, Kayentao K, Traoré MB, Diakité D (2021). Relationship between symptoms, barriers to care and healthcare utilisation among children under five in rural Mali. Trop Med Int Health.

[13] Mochida K, Nonaka D, Wamulume J, Kobayashi J (2021). Supply-side barriers to the use of public healthcare facilities for childhood illness care in rural Zambia: a cross-sectional study linking data from a healthcare facility census to a household survey. Int J Environ Res Public Health.

[14] Young M, Wolfheim C, Marsh DR, Hammamy D (2012). World Health Organization/United Nations Children’s Fund joint statement on integrated community case management: an equity-focused strategy to improve access to essential treatment services for children. Am J Trop Med Hyg.

[15] Moonasar D, Maharaj R, Kunene S, Candrinho B, Saute F, Ntshalintshali N (2016). Towards malaria elimination in the MOSASWA (Mozambique, South Africa and Swaziland) region. Malar J.

[16] Candrinho B, Plucinski MM, Colborn JM, da Silva M, Mathe G, Dimene M (2019). Quality of malaria services offered in public health facilities in three provinces of Mozambique: a cross-sectional study. Malar J.

[17] Davlantes E, Rodrigues EDH, Zulliger R. Mozambique’s Agentes Polivalentes Elementares. CHW Central. 247. 2020. https://chwcentral.org/mozambiques-agentes-polivalentes-elementares/. Accessed 1 June 2022.

